# The Effect of the *Kampo* Medicine Yokukansan on Preoperative Anxiety and Sedation Levels

**DOI:** 10.1155/2014/965045

**Published:** 2014-03-31

**Authors:** Young-Chang Arai, Jun Kawanishi, Yoshikazu Sakakima, Satoshi Sueoka, Akihiro Ito, Yusuke Tawada, Yuki Maruyama, Shinya Banno, Hitomi Takayama, Makoto Nishihara, Takashi Kawai, Tatsunori Ikemoto

**Affiliations:** ^1^Department of Surgery, Toki General Hospital, Gifu 509-5193, Japan; ^2^Multidisciplinary Pain Centre, School of Medicine, Aichi Medical University, 21 Karimata, Nagakutecho, Aichigun, Aichi 480-1195, Japan

## Abstract

*Background*. Preoperative anxiety can lead to unfavorable physiological response such as tachycardia and hypertension. Prevention of preoperative anxiety improves surgical outcome and decreases inpatient stay. Yokukansan is one of prescriptions in *Kampo*, traditional Japanese herbal medicine, and is known to exert anxiolytic effects. The aim of the present study was to compare the effects of diazepam and Yokukansan on preoperative anxiety, salivary amylase activity, and sedation levels. *Methods*. Seventy American Society of Anesthesiologists physical status I or II patients presenting for hemicolectomy under general anesthesia combined with epidural anesthesia were enrolled. The Diazepam group received diazepam 5 mg orally and the Yokukansan group received Yokukansan 2.5 g orally. *Results*. Although levels of anxiety and salivary amylase activity were not different between the two groups, the modified Observer's Assessment of Alertness/Sedation Scale of the Yokukansan group was significantly higher compared to that of the Diazepam group. *Conclusion*. Yokukansan alleviated preoperative anxiety without undesirable sedation, when compared with diazepam.

## 1. Introduction

Preoperative anxiety is a subjective symptom that can induce adverse effects on patients. Excessive anxiety is associated with unfavorable physiological response such as tachycardia and hypertension [1, 2]. And several studies showed that prevention of preoperative anxiety improved surgical outcome and decreased inpatient stay [1, 3]. Moreover, a number of studies have recommended the use of anxiolytic premedication for reducing preoperative anxiety [1, 4]. Although benzodiazepines are effective in reducing preoperative anxiety, these drugs are accompanied by undesirable sedation [5].

*Kampo*, or traditional Japanese herbal medicine based on traditional Chinese herbal medicine, has been used for the treatment of diseases in Japan [6, 7]. Yokukansan is one of the* Kampo* prescriptions for screaming attacks, sleep terrors, and hypnic myoclonia in children [8]. Moreover, it has been used for the treatment of behavioral and psychiatric symptoms of dementia (BPSD) [8–10]. BPSD include delusions, hallucinations, anxiety, sleep problem, and many behavioral problems such as aggressive behavior.

We hypothesized that the use of Yokukansan as premedication alleviates preoperative anxiety without undesirable sedation. In the present study, we therefore compared the effects of diazepam, one of benzodiazepines, and Yokukansan on preoperative anxiety, salivary amylase activity, and sedation levels.

## 2. Methods

After obtaining approval from the Ethics Committees of Aichi Medical University, School of Medicine (a reference number: 13-097), and written informed patient's consent, 70 American Society of Anesthesiologists physical status I or II patients presenting for hemicolectomy under general anesthesia combined with epidural anesthesia were enrolled in the present study from February, 2012, to September, 2013. Patients who had a history of central nervous system or cardiovascular system dysfunction or were allergic to any drugs were not invited to participate in the present study.

A hand-held monitor (COCORO meter, NIPRO, Japan) was used to measure amylase activity in a batch type, using a reagent paper containing 2-chloro-4-nitrophenyl-4-*ο*-*β*-D-galactopyranosylmaltoside (Gal-G2-CNP, Toyobo Co. Ltd., Japan) which is hydrolyzed by amylase [11, 12]. The hydrolyzing reaction goes on until the substrates are completely consumed. The monitor quantifies amylase activity by the assessment of the reaction time. The hand-held monitor consists of a disposable test strip and a monitor. The test strip consists of a collecting paper and a reagent paper for amylase.

The patients were randomly allocated into two groups using computer-generated random numbers: the Diazepam group took diazepam 5 mg and the Yokukansan group received Yokukansan (TJ-54, Tsumura & Co., Tokyo, Japan) 2.5 g. All patients were fasted for 5 h before surgery but were encouraged to take clear fluids until 2 h before induction. One hour and a half hours before anesthesia (one hour and a half hours before the examination of verbal rating scale (VRS) and the modified Observer's Assessment of Alertness/Sedation Scale (OAA/S)), the assigned premedication was given orally. On arrival to the operating room, the collecting paper of a disposable test strip was directly inserted into the oral cavity; approximately 20–30 *μ*L of whole saliva was collected from under the tongue in 30–60 s and then the test strip was set into the monitor for the assessment of salivary amylase activity. Simultaneously, the patients' levels of anxiety and sedation were assessed by investigators, blinded to which drug the patient received, using an 11-point VRS, with 0 = none to 10 = maximum effect [13] and the OAA/S score (Table 1) [14–17], respectively. When measuring the level of sedation by the OAA/S score, responsiveness was the chief characteristic of the four components of the score (a score in the category of responsiveness dominated other categories) (Table 1). That is, if the responsiveness was 5 and the speech, facial expression, and eyes were 5 or less, the level of OAA/S was rated as 5. After collecting these data, anesthesia was induced.

A pilot study of 20 patients showed the mean (SD) of the OAA/S to be 4.5 (0.8) and 4.9 (0.3) in the Diazepam and Yokukansan groups, respectively. We assumed that Yokukansan would improve the OAA/S of at least one degree compared with Diazepam. Thus, the sample size of 30 to 35 was needed to show a difference of 1.0 (SD 1.0) in the OAA/S with a significant level of 0.01 (*α* = 0.01) and a power of 95% (*β* = 0.05). Data are presented as the mean (range), number or the median with the 25th and 75th percentiles. The demographic data and levels of anxiety and salivary amylase activity were analyzed by the Mann-Whitney *U* test. Sex distributions were analyzed by the chi-square test. *P* < 0.05 was considered statistically significant.

## 3. Results

A total of 70 patients were assigned into the Diazepam or Yokukansan group. The two groups were comparable with respect to age, sex distribution, and weight (Table 2).

**Figure 1:**
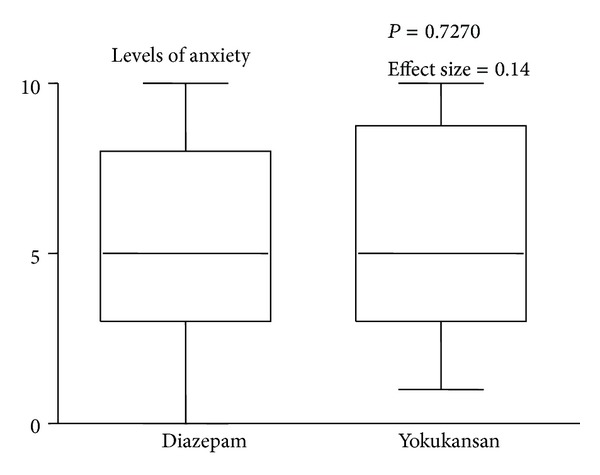
Comparison of anxiety levels assessed by verbal rating scale in Yokukansan and diazepam groups. Horizontal bars represent medians and boxes represent the 25th and 75th percentile ranges. Data were analyzed by the Mann-Whitney *U* test.

**Figure 2:**
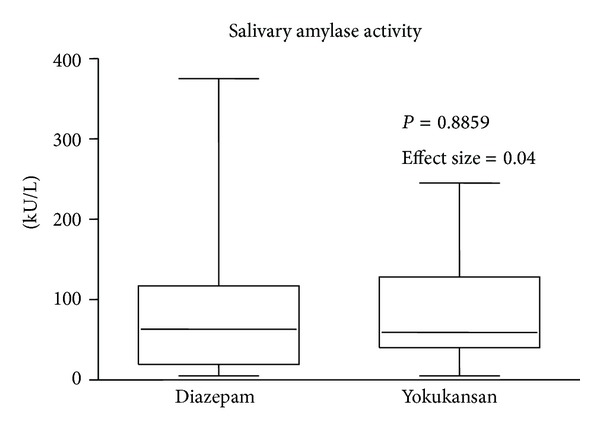
Salivary amylase activity. Horizontal bars represent medians and boxes represent the 25th and 75th percentile ranges. Data were analyzed by the Mann-Whitney *U* test.

**Figure 3:**
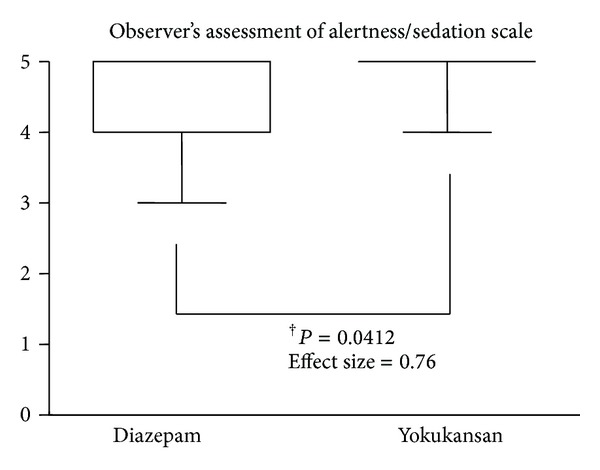
Modified Observer's Assessment of Alertness/Sedation Scale. Horizontal bars represent medians and boxes represent the 25th and 75th percentile ranges. †, different (*P* < 0.05). Data were analyzed by the Mann-Whitney *U* test.

Although levels of anxiety and salivary amylase activity were not different between the two groups (effect size of levels of anxiety: 0.14; effect size of salivary amylase activity: 0.04) (Figures 1 and 2), levels of OAA/S of the Yokukansan group were significantly higher compared to those of the Diazepam group (effect size: 0.76) (Figure 3). No patients experienced any adverse effects.

## 4. Discussion

Preoperative anxiety can lead to unfavorable physiological response such as tachycardia and hypertension [1, 2]. Prevention of preoperative anxiety improves surgical outcome and decreases inpatient stay [1, 3]. Moreover, a number of studies have recommended the use of anxiolytic premedication for reducing preoperative anxiety [1–5, 13]. Although several drugs are effective in reducing preoperative anxiety, these drugs are accompanied by undesirable sedation [5, 13].

Yokukansan is one of the* Kampo* prescriptions and was developed in China in the 16th century as a cure for screaming attacks, sleep terrors, and hypnic myoclonia in children [8, 9]. Behavioral and psychiatric symptoms of dementia (BPSD) occur in patients with different types of dementia and include delusions, hallucinations, anxiety, sleep problem, and many behavioral problems such as aggressive behavior. Yokukansan has been used for the treatment of BPSD [8–10]. Yokukansan is known to work against glutamate-mediated excitotoxicity [9]. Also, it is known to exert anxiolytic effects via serotonin receptors [8].

Evaluation of psychological stress has been relying on subjective assessment tools such as state-trait anxiety inventory score or visual analog scale. However, if subjects fail to reliably report, subjective evaluation tools are not reliable [11, 12]. Recently, measurement of salivary biomarkers, which are sampled noninvasively, was evaluated as stress biomarkers [11]. Especially, salivary *α*-amylase is associated with changes in plasma noradrenaline and is utilized as an excellent index for psychological stress [11, 12]. Thus, we evaluated psychological stress by not only a subjective assessment tool but also by salivary *α*-amylase activity. In the present study, without undesirable sedation, Yokukansan 2.5 g showed the same anxiolytic effect as diazepam 5 mg showed.

There is a limitation in the present report. Although the patients' weight varied from 36 to 109 kg in the present study, we did not observe that the weight diversity influenced the effectiveness of diazepam or Yokukansan. However, we need to further investigate the effect of Yokukansan per weight.

In conclusion, Yokukansan alleviated preoperative anxiety without undesirable sedation when compared with diazepam.

## Figures and Tables

**Table 1:** Modified Observer's Assessment of Alertness/Sedation Scale (OAA/S). In assessing the OAA/S score the main criterion was responsiveness.

Score	Responsiveness	Speech	Facial expression	Eyes
5 (Alert)	Responds readily to voice with normal tone	Normal	Normal	Clear, no ptosis
4	Responds slowly to voice with normal tone	Mild slowing	Mild relaxation	Mild ptosis (less than half the eye)
3	Responds after calling loudly or repeatedly	Prominent slowing or slurring	Marked relaxation (slack jaw)	Marked ptosis (half the eye or more)
2	Responds after mild prodding or shaking	Few recognizable words	—	—
1	Does not respond to mild prodding or shaking	—	—	—
0	Does not respond to pain	—	—	—

**Table 2:** Patient's characteristics.

	Diazepam	Yokukansan	*P*
Age (years)*	66 [35–85]	63 [30–85]	0.4013
Sex (M/F)**	23/11	24/12	0.9475
Weight (kg)*	59 [36–109]	61 [35–83]	0.5990

Values are numbers or median [range]. *Mann-Whitney *U* test. **Chi-square test.
